# An alternative synthesis of Vandetanib (Caprelsa™) *via* a microwave accelerated Dimroth rearrangement

**DOI:** 10.1016/j.tetlet.2017.02.082

**Published:** 2017-04-12

**Authors:** Kayleigh L. Brocklesby, Jennifer S. Waby, Chris Cawthorne, Graham Smith

**Affiliations:** a Hull-York Medical School, University of York, Heslington, York YO10 5DD, UK; b Division of Radiotherapy and Imaging, Institute of Cancer Research, London SW7 3RP, UK; c School of Biological, Biomedical and Environmental Sciences, University of Hull, Cottingham Road, Hull HU6 7RX, UK; d PET Research Centre, University of Hull, Cottingham Road, Hull HU6 7RX, UK

**Keywords:** Vandetanib, Quinazoline, Dimroth rearrangement, Tyrosine kinase inhibitor

## Abstract

Vandetanib is an orally available tyrosine kinase inhibitor used in the treatment of cancer. The current synthesis proceeds *via* an unstable 4-chloroquinazoline, using harsh reagents, in addition to requiring sequential protection and deprotection steps. In the present work, use of the Dimroth rearrangement in the key quinazoline forming step enabled the synthesis of Vandetanib in nine steps (compared to the previously reported 12–14).

## Highlights

• Synthesis of Vandetanib achieved in fewer steps (9 versus the previous best of 12).
• New synthetic route has fewer steps that require chromatographic purification.
• Key step is a microwave assisted Dimroth rearrangement to provide the core quinazoline.

## Graphical abstract

**Figure:**
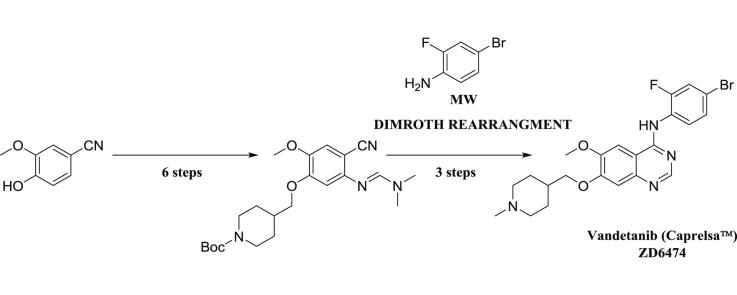

## Introduction

**Fig. 1:**
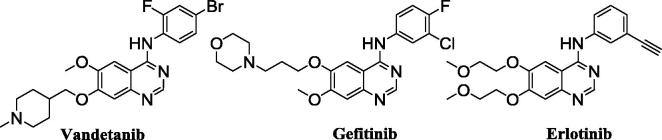
Structures of Vandetanib (Caprelsa™), Gefitinib (Iressa™) and Erlotinib (Tarceva™).

Vandetanib (Caprelsa™), discovered by AstraZeneca, is an orally available tyrosine kinase inhibitor with activity against VEGFR2/EGFR/RET which is currently used in the treatment of medullary thyroid cancer (Fig. 1).^1^ Vandetanib is representative of a wide class of 4-anilinoquinazoline drug molecules that function as adenosine mimetics and bind at the tyrosine kinase intracellular receptor site; other examples of this compound class include Erlotinib and Gefitinib (Fig. 1).^1^, ^2^ As part of a programme of work investigating the use of tyrosine kinase inhibitors (TKI’s) for application to Positron Emission Tomography (PET) imaging of receptor expression, the improved synthesis of Vandetanib and related compounds was investigated.

**Scheme 1:**
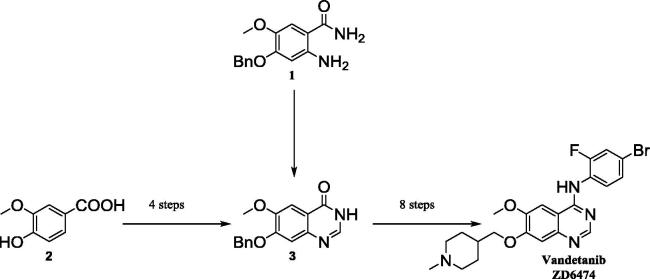
Current synthetic procedure to Vandetanib *via* the key quinazolinone intermediate **3.**

The current literature precedence for the synthesis of Vandetanib *via* the quinazolinone intermediate **3** is summarised in Scheme 1 (shown in more detail in ESI, Scheme S1). This is a time-consuming synthetic strategy involving twelve steps overall and harsh reagents/reaction conditions. The original synthesis utilised amide **1**,^3^ which requires pre-preparation due to a lack of commercial availability and Gold’s reagent ([3-(dimethylamino)-2-azaprop-2-en-1-ylidene]dimethylammonium chloride) to directly construct **3** (Scheme 1).^4^, ^5^, ^6^ More recent patents have avoided the use of Gold’s reagent and proceeded *via* benzoic acid **2**; however, this still involves progression through multiple protection and deprotection steps.^4^ The current work reports a new and improved synthesis of Vandetanib, avoiding quinazolinone **3** and utilising the Dimroth rearrangement for a streamlined synthetic procedure.^7^, ^8^, ^9^, ^10^

## Results and discussion

**Scheme 2:**
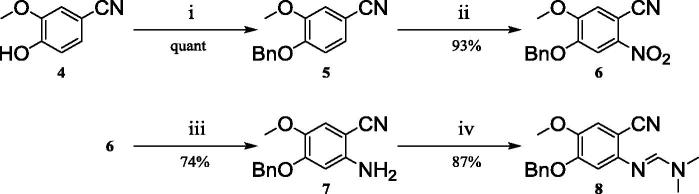
Reagents and conditions: i) Benzyl bromide, K_2_CO_3_, DMF, rt, 18 h; ii) Nitric acid, Ac_2_O, 0 °C to rt, 18 h; iii) NaHCO_3_, tetra-butyl ammonium chloride, Sodium dithionite, DCM, water, rt, 2 h; iv) DMF-DMA, microwave, 90 °C, 15 min.

The synthesis commenced from the inexpensive building block 4-hydroxy-3-methoxybenzonitrile **4** (Scheme 2), which was alkylated with benzyl bromide in quantitative yield without the need for extensive purification.^11^ Nitration of **5** under mild conditions afforded **6** in 93% yield as a yellow precipitate which required no further purification.

Literature precedence for the reduction of **6** using iron and ammonium formate, revealed a difficult work-up associated with poor yields;^12^ alternative literature procedures suggested the use of sodium dithionite.^13^ Pleasingly, reduction using sodium dithionite gave **7** in 74% yield, with no purification required (Scheme 2). The synthesis of **8** (Scheme 2 and Table 1) was troublesome due to the poor solubility of aniline **7** in organic solvents. Ultimately the reaction was performed neat with a large excess of DMF-DMA to aid solubility and formation of the formamidine; toluene was also tested as a co-solvent with no observable effect on the reaction progress. Microwave irradiation for 15 minutes at 90 °C (Entry 1) afforded only minimal conversion to formamidine **8**; however, extension of the reaction time by a further 15 minutes (to a total of 30 minutes) resulted in an improved yield of 95% (Entry 5) due to the increased time for solubilisation in DMF-DMA to occur. Conventional heating (Entry 2) at the same temperature provided **8** in a reduced yield of 58% despite extending the reaction time to 120 minutes. Of note, on smaller scales chromatographic removal of DMF-DMA was required, but on larger scales (e.g. 8 mmol) formamidine **8** was observed to precipitate from DMF-DMA.

**Scheme 3:**
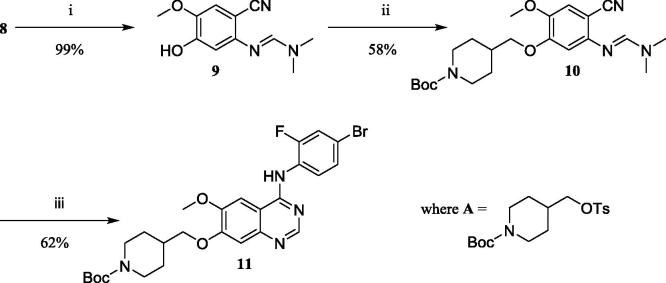
Reagents and conditions: i) TFA, microwave (max. power 400 W), 70 °C, 45 min; ii) **A**, Cs_2_CO_3_, MeCN, reflux, 3 hr; iii) 4-bromo-2-fluoroaniline, 130 °C, 1 h.

TFA mediated debenzylation of **8** was selected owing to the potential vulnerability to hydrogenation induced reduction of the formamidine, and afforded **9** in an almost quantitative yield. Alkylation of **9** afforded **10** in 58% yield using *tert*-butyl-4-(tosyloxy)methyl)piperidine-1-carboxylate (**A** in Scheme 3), which was synthesised according to a literature method.^14^

**Scheme 4:**
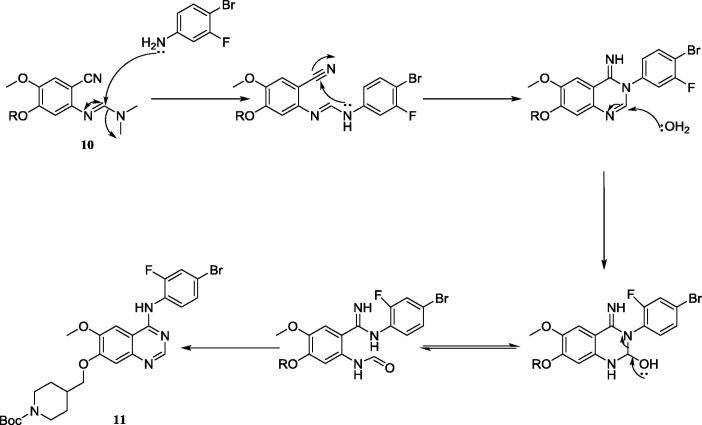
Proposed mechanism for the Dimroth rearrangement; derived from Chandregowdra and co-workers in their synthesis of Erlotinib and Gefitinib.^16^

The key Dimroth rearrangement step was then investigated commencing from **10** in the presence of 4-bromo-3-fluoroaniline (1 equiv.) and acetic acid with microwave heating at 130 °C for 45 min. No product was observed by either TLC or LCMS when irradiated for 15 minutes at 118 °C; this contrasted with previous reports on the synthesis of related 4-anilinoquinazolines such as Gefitinib.^15^, ^16^ Duration of the microwave irradiation was irrelevant for the synthesis of **11**, which was temperature dependent and required 130 °C for complete conversion, highlighting the possible inactivated nature of the reagents. Quinazoline **11** was isolated in 62% yield after chromatographic purification. The proposed mechanism for this rearrangement is illustrated in Scheme 4.

**Scheme 5:**
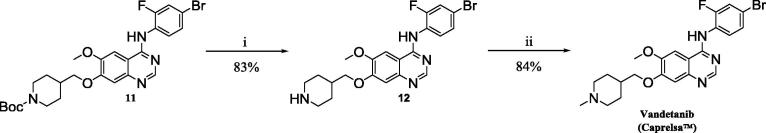
i) TFA, DCM, rt, 2 h; ii) HCHO, AcOH, NaBH(OAc)_3_, MeOH, DCM, rt, 2 h.

The penultimate step in the synthesis of Vandetanib was the BOC deprotection of piperidine **11**. Deprotection according to literature procedures resulted in low yields during aqueous work-up (Scheme 5).^3^ However, through following the literature deprotection procedure^4^ and avoiding aqueous work-up, direct silica chromatography purification afforded **12** in 83% yield. Reductive amination with formaldehyde according to the literature procedure^4^ yielded Vandetanib (Caprelsa™) in 84% yield, an overall 7% yield over nine steps.

## Conclusion

In this report we have presented an alternative route to the synthesis of Vandetanib *via* the Dimroth rearrangement. Ultimately this new synthetic procedure is a reduction of three steps compared to the most concise published method and avoids the series of protection and deprotection steps currently established by several literature procedures. Chromatography is only required for four steps in this synthesis compared to multiple steps in the previous reports. Overall Vandetanib was synthesised in 7% yield over 9 steps compared to 4–20% yield over 12–14 steps in the previously reported methods.

## Experimental

Experimental data can be found in the Supplementary data file associated with this report. In addition to experimental information and characterisation data this file also contains NMR spectra for selected compounds.

## Figures and Tables

**Table 1:** Optimisation for the synthesis of **8**.

Entry	Solvent	Time (min)	Heating (temp(°C))	Work-up	Yield
**1**	DMF-DMA	15	Microwave (90 °C)	A	2%
**2**	DMF-DMA-toluene	120	Conventional (185 °C)^∗^	None	58%
**3**	DMF-DMA-toluene	30	Microwave (90 °C)	A	95%
**4**	DMF-DMA-toluene	30	Microwave (90 °C)	B	94%
**5**	DMF-DMA	30	Microwave (90 °C)	A or B	94–95%

A) Allowed to precipitate overnight; B) Removal of DMF-DMA using silica chromatography; ^∗^Distillation facilitated removal of DMF-DMA from the product. Reagents and conditions: **7** (500 mg, 1.97 mmol), either DMF-DMA (3 mL) or DMF-DMA in toluene (1:1, total volume 3 mL); microwave reactions were conducted in a Biotage Initiator with a maximum power output of 400 W.
