# Supplementary material for: An alternative synthesis of Vandetanib (Caprelsa™) via a microwave accelerated Dimroth rearrangement
Source: Tetrahedron Lett. 2017 Apr 12;58(15):1467–9. doi: 10.1016/j.tetlet.2017.02.082 (PMC5381755; doi:10.1016/j.tetlet.2017.02.082)
Supplement: Supplementary data [file mmc1.docx]

An alternative synthesis of Vandetanib (Caprelsa™) *via* a microwave accelerated Dimroth rearrangement

Kayleigh L. Brocklesby^1,2^, Jennifer S. Waby^3§^, Chris Cawthorne^4^, Graham Smith^2^*

^1^Hull-York Medical School, University of York, Heslington, York YO10 5DD; ^2^Division of Radiotherapy and Imaging, Institute of Cancer Research, London, SW7 3RP, UK; ^3^School of Biological, Biomedical and Environmental Sciences, University of Hull, Cottingham Road, Hull, HU6 7RX, UK. ^4^PET Research Centre, University of Hull, Cottingham Road, Hull, HU6 7RX, UK

* Email: [graham.smith@icr.ac.uk](mailto:graham.smith@icr.ac.uk); Tel +44 (0)2087224482

^§^Present address: Faculty of Life Sciences, Richmond Building Room H15, University of Bradford, Bradford, West Yorkshire, BD7 1DP, UK.

**Supporting information**

| 1. **Literature routes to Vandetanib** | **S2** |
| --- | --- |
| 1. **General Information** | **S5** |
| 1. **Synthetic procedures** | **S7** |
| 1. **NMR Spectra** | **S17** |

1. Literature routes to Vandetanib (Caprelsa™)

Synthesis 1^1-3^

Scheme 1: Synthesis *via* carboxamide 1 and quinazoline intermediate 3 i) BnCl, K_2_CO_3_, EtOH reflux, 2.5d; ii) HNO_3_,0°C–rt, 1 hr^;^ iii) KMnO_4_, Acetone 50°C, 40 min iv) SOCl_2_, reflux, 22 hr^;^ v) conc NH_3_, dioxane, rt, 2 hr; vi) Fe, AcOH, 90 °C, 65 min; vii) Gold`s Reagent, dioxane, sodium acetate, acetic acid, reflux, 27 hr; viii) SOCl_2_, DMF, reflux, 1 hr; ix) 4-bromo-2-fluoroanline, IPA, reflux, 4 hr; x) TFA, reflux, 1 hr; xi) tert-Butyl-4-(tosyloxy)methyl)piperidine-1-carboxylate A, K_2_CO_3_, DMF, 95 °C, 2 hr; xii) TFA, DCM, rt, 1 hr; xiii) formaldehyde, Na(CH₃COO)₃BH, rt, 2 hr.

Synthesis 2^4^

Scheme 2: Literature synthesis of key quinazoline intermediate 3 i) BnBr, DIPEA, MeCN, reflux-rt, 48 hr; ii) AcOH, H_2_SO_4_, HNO_3_, 10-40 °C, 26 hr^;^ iii) Sodium dithionite, MeCN, H_2_O, rt-65-0 °C, 3 hr; iv) formamidine acetate, isobutanol, 95 °C for 6 hr then rt for 90 min; v) BnBr, K_2_CO_3_, DMF, 100 °C, 3 hr; vi) AcOH, HNO_3_10-40 °C, 26 hr^;^ vii) Fe, NH_4_OAc, H_2_O, toluene, reflux, 1 hr; vii) formamide, ammonium acetate, 180 °C, 2 hr.

Synthesis 3^2, 3^

Scheme 3: Continued synthesis from quinazoline 3, according to the AstraZeneca publications i) NaH, POM-Cl, DMF, 20 °C, 4 hr; ii) Pd/C, H_2_, AcOH, rt, 40 min; iii) tert-Butyl-4-(tosyloxy)methyl)piperidine-1-carboxylate A, K_2_CO_3_, DMF, 100 °C, 2.5 hr; iv) TFA, DCM, rt, 1 hr v) HCHO, NaBH_3_CN, MeOH, THF, rt, 30 min; vi) NH_3_, MeOH, rt, 20 hr; vii) SOCl_2_, DMF, 85 °C, 1 hr; viii) 4-bromo-2-fluoroanline, IPA, HCl, reflux, 90 min

1. General Information

Unless otherwise stated, reagents and solvents were purchased from commercial suppliers (Sigma Aldrich, Apollo Scientific, Fisher, Acros Organic and Alfa Aesar) and used without further purification. *N,N*-Dimethylformamide dimethyl acetal (DMF-DMA) was purchased from Sigma Aldrich as an undefined mixture. Chromatography solvents were HPLC grade and also used without further purification. All solvent mixtures are quoted as volumes prior to mixing (v/v). For microwave reactions a Biotage Initiator with a maximum power setting of 400W was used.

*NMR*

^1^H NMR spectra were recorded on a Bruker Advance 500 MHz spectrometer using an internal deuterium lock or a JEOL-ECP 400 MHz FT-NMR. Chemical shifts (δ) are measured in parts per million (ppm) were referenced to the following residual solvent peaks: CHCl_3_ (δ 7.26), DMSO (δ 2.5) or MeOH (δ 3.35). Compound assignments were aided by 2D NMR techniques; COSY, HSQC, DEPT and NOSEY. Data is presented in the following format: chemical shift (multiplicity, integration, coupling constant (J in Hz)). Mulitplicities are quoted as following: s (singlet), brs (broad singlet), d (doublet), t (triplet), q (quartet), quin (quintet), dd (doublet of doublets), dt (doublet of triplets), ddd (doublet of doublet of doublet) and m (multiplet). All aromatic doublets are assumed to be true doublets.

^13^C NMR spectra were recorded on a Bruker Advance 500 MHz spectrometer using an internal deuterium lock or a JEOL-ECP 400 MHz FT-NMR. Chemical shifts (δ) are measured in parts per million (ppm) relative to tetramethylsilane (TMS) and were referenced to the following residual solvent peaks: CHCl_3_ (δ 77.23) or DMSO (δ 39.51). Data is presented in the following format: chemical shift (assignment). Where it is the case splitting is caused by fluorine, Data is presented in the following format: chemical shift (multiplicity, coupling constant (J in Hz)).

^19^F NMR decoupled spectra were recorded on a Bruker Advance 500 MHz spectrometer and were referenced to an external standard of CFCl_3_ (neat) set to δ=0 ppm.

*HRMS and Purity*

High resolution mass spectrometry (HRMS) was performed on an Agilent 1200 series HPLC system, with diode array detector operating at 254 nm, fitted with a Merck Chromolith SpeedROD RP-18e 50 × 4.6 nm column at a temperature of 22 °C, connected to a Agilent 6520 Quadrupole Time Of Flight (QToF) mass spectrometer (simultaneous ESI and APCI). The following solvent system, at a flow rate of 2 mL min^-1^ was used: solvent A: Methanol: solvent B: 0.1% formic acid in water. Gradient elution was as follows: 1: (A: B) to 9:1 (A: B) for 1 minute, then reversion back to 1:9 (A:B) over 0.3 minutes, 1:9 (A:B) for 0.2 minutes.

Purity of compounds (at a concentration of 1 µM) are indicated by HPLC purity as described above (Method A) or on an Agilent 1200 series (Phenomenex Luna® 5 µm C18(2) 100 Å, LC Column 150 × 4.6 mm) utilising the following gradient system at 1 mL min^-1^ (2). Solvent A: Water and 10 mM NaH_3_PO_4_ Solvent B: Acetonitrile and 0.1% H_3_PO_4_. Gradient elution was as follows: 80:20 (A:B) to 55:45 (A:B) for 5 minutes, then to 5:95 (A:B) in 10 minutes and held for a further 5 minutes, followed by reversion back to 80:20 (A:B) over 1 minute and held for a further 9 minutes (Method B).

All purities are expressed as a percentage (%).

1. Synthetic Procedures

**4-Benzyloxy-3-methoxybenzonitrile^5^ (5)**

4-Hydroxy-3-methoxybenzonitrile (3.4 g, 22.8 mmol) was dissolved in DMF (45 mL), potassium carbonate (4.73 g, 34.2 mmol) was added slowly, followed by benzyl bromide (3.0 mL, 25.1 mmol). The reaction mixture was stirred overnight at ambient temperature then brine (50 mL) solution was added. The precipitate was collected affording the title compound as a white solid (5.45 g, 100%).

Mp: 81-83 °C (lit 81-83 °C^6^); ^1^H NMR (500 MHz, CDCl_3_) δ: 3.91 (s, 3H, OC*H*_3_), 5.21 (s, 2H, Ar-C*H*_2_), 6.92 (d, 1H, Ar*H*, *J =* 8.3 Hz), 7.11 (d, 1H, Ar*H*, *J =* 1.9 Hz), 7.22 (dd, 1H, Ar*H*, *J* = 1.9, 8.3 Hz), 7.30-7.46 (m, 5H, Ar*H*). ^13^C NMR (125 MHz, CDCl_3_) δ: 56.20, 70.89, 104.18, 113.32, 114.40, 119.23, 126.27, 127.25, 128.77, 128.80, 135.81, 149.68, 152.00. HRMS (ESI, m/z): calc’d for C_15_H_14_NO_2_ [M+H]^+^ 240.1019 found: 240.1017

**4-Benzyloxy-5-methoxy-2-nitrobenzonitrile^7^ (6)**

4-Benzyloxy-3-methoxybenzonitrile (3.5 g, 14.62 mmol) was dissolved in acetic anhydride (40 mL), nitric acid (3.6 mL, 65 mmol) was added slowly over a 30 minute period and then stirred overnight. The reaction was poured onto ice-water (25 mL) and the precipitate collected, washed with water affording the title compound as a yellow solid (3.87 g, 93%).

Mp: 159-162 °C; ^1^H NMR (500 MHz, *d_6_*-DMSO) δ: 3.97 (s, 3H, OC*H*_3_), 5.33 (s, 2H, ArC*H*_2_), 7.38 (ddd, 2H, Ar*H*, *J =* 2.0, 4.9, 8.4 Hz), 7.42(ddd, 2H, Ar*H*, *J =* 2.7, 4.5, 10.7 Hz) , 7.48(dd, 1H, Ar*H*, *J =* 0.8, 7.7 Hz) , 7.72 (s, 1H, Ar*H*), 8.01 (s, 1H, Ar*H*). ^13^C NMR (125 MHz, *d_6_*-DMSO) δ: 57.60, 71.32, 100.29, 110.06, 116.20, 117.17, 128.63, 128.88, 129.07, 135.90, 142.61, 151.24, 153.93. HRMS (ESI, m/z): calc’d for C_15_H_13_N_2_O_4_ [M+H]^+^ 285.087 found: 285.0888

**2-Amino-4-benzyloxy-5-methoxybenzonitrile^8^ (7)**

4-Benzyloxy-5-methoxy-2-nitrobenzonitrile (3.46 g, 12.18 mmol), NaHCO_3_ (0.22 g, 2.03 mmol) and *tert*-butylammonium chloride (2.25 g, 8.1 mmol) was dissolved in DCM (41 mL) and water (56 ml). The solution was stirred vigorously and sodium dithionite (4.93 g, 28.5 mmol) was added over a two hour period. The solution was stirred for a further hour and extracted. The aqueous phase was extracted twice with DCM (50 mL) and the combined organic layer washed with water (75 mL). The organic layer was dried over MgSO_4_ and concentrated to a quarter of the initial volume. HCl in dioxane (0.6 mmol) and ether was added and cooled to 0 °C, the precipitate was collected and washed with diethyl ether. The residue was re-suspended in methanol (45 mL) and basified with saturated NaHCO_3_ to pH 8. The solid was collected to afford the title compound as a yellow solid (2.29 g, 74%).

Mp: 184-186 °C; ^1^H NMR (500 MHz, *d_6_*-DMSO) δ: 3.66 (s, 3H, OC*H*_3_), 5.05 (s, 2H, ArC*H*_2_), 5.62 (brs, 2H, N*H*_2_), 6.51 (s, 1H, Ar*H*), 6.91 (s, 1H, Ar*H*), 7.33-7.37 (m 1H, Ar*H*), 7.40 (ddd, 2H, Ar*H*, *J =* 0.9, 6.8, 7.9 Hz), 7.42-7.46 (m, 2H, Ar*H*). ^13^C NMR (125 MHz, *d_6_*-DMSO) δ: 56.71, 70.01, 84.32, 100.64, 114.77, 119.13, 128.40, 128.54, 128.94, 136.70, 140.90, 148.69, 153.84. HRMS (ESI, m/z): calc’d for C_15_H_15_N_2_O_2_ [M+H]^+^ 255.1128 found: 255.1124

**(*E*)-*N*`-(5-Benzyloxy-2-cyano-4-methoxyphenyl)-*N,N*-dimethylformimidamide^9^ (8)**

2-Amino-4-benzyloxy-5-methoxybenzonitrile (500 mg, 1.97 mmol) was dissolved in DMF-DMA (326 µL, 2.46 mmol) and irradiated for 15 minutes at 90 °C. The solution was purified by column chromatography (2:8 EtOAc: DCM) to afford the title compound as an orange solid (528 mg, 87%).

Mp: 130-132 °C (lit 131-133 °C^9^); ^1^H NMR (500 MHz, *d_6_*_-_DMSO) δ: 2.96 (s, 3H, NC*H*_3_), 3.06 (s, 3H, NC*H*_3_), 3.73 (s, 3H, OC*H*_3_), 5.15 (s, 2H, ArC*H*_2_), 6.88 (s, 1H, Ar*H*), 7.12 (s, 1H, Ar*H*), 7.34-7.38 (m, 1H, Ar*H*), 7.39-7.44 (m, 2H, Ar*H*), 7.44-7.48 (m, 2H^,^ Ar*H*), 7.90 (s, 1H, NC*H*N). ^13^C NMR (125 MHz, *d_6_*-DMSO) δ: 34.41, 40.37, 56.49, 70.40, 96.61, 104.41, 115.01, 119.51, 128.52, 128.57, 128.98, 136.85, 144.60, 150.93, 152.88, 155.01. HRMS (ESI, m/z): calc’d for C_18_H_20_N_3_O_2_ [M+H]^+^ 310.155 found: 310.1544

**(*E*)-*N*`-(2-Cyano-5-hydroxy-4-methoxyphenyl)-*N,N*-dimethylformimidamide^9^ (9)**

(*E*)-*N*`-(5-Benzyloxy-2-cyano-4-methoxyphenyl)-*N,N*-dimethylformimidamide (100 mg, 0.323 mmol) was dissolved in TFA (1.29 mL) and irradiated for 45 minutes at 70 °C. The reaction mixture was concentrated under reduced pressure and dissolved in DCM (10 mL) and washed with saturated NaHCO_3_ (10 mL), dried over MgSO_4_ and concentrated under reduced pressure to afford the title compound as a yellow solid (70 mg, 99%).

Mp: 109-111 °C; ^1^H NMR (500 MHz, *d_6_*-DMSO) δ: 2.93 (s, 3H, NC*H*_3_), 3.02 (s, 3H, NC*H*_3_), 3.74 (s, 3H, OC*H*_3_), 6.50 (s, 1H, Ar*H*), 7.08 (s, 1H, Ar*H*), 7.76 (s, 1H, NC*H*N), 9.84 (s, 1H, O*H*). ^13^C NMR (125 MHz, *d_6_*-DMSO) δ: 34.44, 40.03, 56.60, 95.49, 106.63, 115.57, 119.81, 143.70, 148.64, 152.28, 154.68. HRMS (ESI, m/z): calc’d for C_11_H_14_N_3_O_2_ [M+H]^+^ 220.1081 found: 220.1076

**(*E*)-*tert*-Butyl-4-((4-cyano-5-(((dimethylamino)methylene)amino)-2-methoxyphenoxy)methyl)piperidine-1-carboxylate^10^ (10)**

(*E*)-*N*`-(2-Cyano-5-hydroxy-4-methoxyphenyl)-*N,N*-dimethylformimidamide (863 mg, 3.94 mmol), *tert*-butyl-4-(tosyloxy)methyl)piperidine-1-carboxylate **A** (1.6 g, 4.33 mmol) and caesium carbonate (3.2 g, 9.85 mmol) was dissolved in MeCN (25 mL) and heated at reflux for three hours. The reaction mixture was concentrated under reduced pressure, then stirred in saturated NaHCO_3_ solution (30 mL) and extracted with EtOAc (3 × 50 mL). The organic layers were combined and dried over MgSO_4_ and concentrated under reduced pressure. Column chromatography purification (1:1 to 100% EtOAc: hexane) afforded the title compound as a colourless oil which solidified on standing (853 mg, 58%).

^1^H NMR (500 MHz, *d_6_*_-_DMSO) δ: 1.10-1.18 (m, 2H, C*H*_2_), 1.40 (s, 9H, CC*H*_3_), 1.68 (d, 2H, C*H*_2_, *J =* 10.9 Hz), 2.03 (m, 1H, CC*H*), 2.92 (m, 2H, C*H*_2_) 2.95 (s, 3H, NC*H*_3_), 3.05 (s, 3H, NC*H*_3_), 3.18 (m, 2H, C*H*_2_), 3.72 (s, 3H, OC*H*_3_), 3.89 (d, 2H, OC*H*_2_C, *J =* 6.5 Hz), 6.72 (s, 1H, Ar*H*), 7.08 (s, 1H, Ar*H*), 7.88 (s, 1H, NC*H*N). ^13^C NMR (125 MHz, *d_6_*-DMSO) δ: 14.55, 21.22, 28.56, 28.78, 34.40, 35.72, 39.74, 40.49, 56.58, 60.21 72.82, 78.98, 96.36, 103.91, 115.06, 119.56, 144.53, 153.31, 154.40, 154.97. HRMS (ESI, m/z): calc’d for C_22_H_33_N_4_O_4_ [M+H]^+^ 417.2496 found: 417.2507

***tert*-Butyl-4-(tosyloxy)methyl)piperidine-1-carboxylate^11^ (A)**

*tert*-Butyl-4-(hydroxymethyl)piperidine-1-carboxylate (1 g, 4.64 mmol) was stirred in pyridine (3.7 mL) at 0 °C. *p*-Toluene sulfonyl chloride (0.974 mg, 5.11 mmol) was added in one batch under nitrogen and the mixture stirred for 100 minutes at 0 °C. The mixture was allowed to warm to ambient temperature and stirred overnight. The mixture was poured onto water (25 mL) and extracted with ethyl acetate (3 × 15 mL). The organic layer was washed with 1M HCl (15 mL) and brine (15 mL), dried over MgSO_4_ and concentrated under reduced pressure. Purification by column chromatography (1:20 to 1:1 EtOAc: hexane) afforded *tert*-butyl-4-(tosyloxy)methyl)piperidine-1-carboxylate as a colourless oil which solidified on standing (0.981 g, 57%).

^1^H NMR (500 MHz, CDCl_3_) δ: 1.11 (dtd, 2H, C*H*_2_, *J* = 4.9, 10.0, 13.2 Hz), 1.42-1.52 (m, 9H, C*H*_3_), 1.61-1.69 (m, 2H, C*H*_2_), 1.83 (dddd, 1H, C*H*, *J =* 2.4, 5.0, 10.0, 11.8 Hz), 2.47 (s, 3H, C*H*_3_), 2.61-2.73 (m, 2H, C*H*_2_), 3.86 (d, 2H, C*H*_2_, *J* = 6.5 Hz), 7.36 (d, 2H, Ar*H*, *J =* 8.0 Hz), 7.79 (d, 2H, Ar*H*, *J* = 8.0 Hz). ^13^C NMR (125 MHz, CDCl_3_) δ: 14.20, 21.66, 28.42, 35.78, 43.16, 73.99, 79.55, 127.88, 129.89, 132.91, 144.86, 154.67. HRMS (ESI): calc’d for C_18_H_27_NO_5_SNa [M+Na]^+^ 392.1502; found 392.1479

***tert*-Butyl 4-(((4-((4-bromo-2-fluorophenyl)amino)-6-methoxyquinazolin-7-yl)oxy)methyl)piperidine-1-carboxylate^2, 12^ (11)**

(*E*)-*tert*-Butyl-4-((4-cyano-5-(((dimethylamino)methylene)amino)-2 methoxyphenoxy)methyl)piperidine-1-carboxylate (220 mg, 0.528 mmol) was combined with 4-bromo-2-fluoroaniline (101 mg, 0.528 mmol) in acetic acid (1 mL) and subjected to microwave irradiation for one hour at 130 °C. The reaction mixture was loaded directly onto a column (1:1 EtOAc: hexane) to afford the title compound as a white solid (135 mg, 62%).

Mp: 222-224 °C (lit 223-225 °C^12^); ^1^H NMR (500 MHz, *d_6_*-DMSO) δ: 1.14-1.28 (m, 4H, C*H*_2_), 1.41 (s, 9H, CC*H*_3_), 1.79 (d, 2H, C*H*_2_, *J =* 11.3 Hz), 1.99-2.07 (m, 2H, C*H*_2_), 2.77-2.86 (m, 1H, C*H*_2_), 3.32 (s, 2H, C*H*_2_), 3.94 (s, 3H, OCH_3_) 4.02 (d, 2H, OC*H*_2,_ *J* = 6.1 Hz), 7.19 (s, 1H, Ar*H*), 7.47 (dd, 1H, Ar*H, J =* 1.9, 8.8 Hz), 7.53 (t, 1H, Ar*H*, *J =* 8.4 Hz), 7.66 (dd, 1H, Ar*H*, *J =* 2.2, 10.0 Hz), 7.79 (s, 1H, Ar*H*), 8.35 (s, 1H, Ar*H*), 9.53 (s, 1H, N*H*). ^13^C NMR (125 MHz, *d_6_*-DMSO) δ: 28.58, 35.55,, 56.63, 72.89, 78.99, 102.43, 108.19(d, *J =* 28.9 Hz), 109.05, 111.61, 119.70, 119.89, 123.51, 126.92, 127.95, 130.01 (d, *J =* 27.2 Hz), 147.40, 149.54, 153.40, 154.17, 154.39 (d, *J =* 125 Hz), 157.34. ^19^F (471 MHz, *d_6_*-DMSO) δ: -115.56 (t, *J =* 9.0 Hz). HRMS (ESI, m/z): calc’d for C_26_H_32_N_­4_O_4_FBr [M+H]^+^ 561.1507 found: 561.1511

***N*-(4-Bromo-2-fluorophenyl)-6-methoxy-7-(piperidin-4-ylmethoxy)quinazolin-4-amine^2, 12^ (12)**

(*E*)-*tert*-Butyl-4-((4-cyano-5-(((dimethylamino)methylene)amino)-2-methoxyphenoxy)methyl)piperidine-1-carboxylate (600 mg, 1.07 mmol), was dissolved in DCM (1 mL) and TFA (0.3 mL) was added, the solution was stirred at ambient temperature for two hours. The reaction mixture was then concentrated under reduced pressure and loaded directly onto a column (0-1:100-5 MeOH: DCM), affording the title compound as a colourless solid (256 mg, 83%).

Mp: 221-223 °C (lit 221-223 °C^12^); ^1^H NMR (500 MHz, *d_6_*_-_DMSO) δ: 1.33 (dd, 2H, CH_2_, *J =* 6.7,11.8 Hz), 1.82 (d, 2H, C*H*_2_, *J =* 11.9 Hz), 2.01 (brs, s, 1H, CC*H*), 2.66 (t, 2H, C*H*_2_, *J =* 15.5 Hz), 3.11 (d, 2H, C*H*_2_, *J =* 11.6 Hz), 3.94 (s, 3H, OC*H*_3_) , 4.01(d, 2H, OC*H*_2_, *J =* 6.5 Hz), 7.20 (s, 1H, Ar*H*), 7.47 (d, 1H, Ar*H, J =* 8.6 Hz), 7.53 (t, 1H, Ar*H, J =* 8.3 Hz), 7.67 (dd, 1H, Ar*H, J =* 1.8, 9.9 Hz), 7.81 (s, 1H, Ar*H*), 8.35 (s, 1H, Ar*H*), 9.56 (s, 1H, N*H*). ^13^C NMR (125 MHz, *d_6_*-DMSO) δ: 28.68, 34.92, 46.98, 49.32, 55.69, 70.65, 79.86, 102.82, 107.52 (d, *J =* 27.6 Hz), 108.91, 114.21, 114.74, 117.65, 121.21, 127.68, 131.32 (d, *J =* 28.3 Hz), 153.04, 155.23, 155.99 (d, *J =* 132.3 Hz), 157.08, 158.86, 164.06^19^F (471 MHz, *d_6_*-DMSO) δ: -116.99 (m, 1F). HRMS (ESI, m/z): calc’d for C_22_H_23_N_4_O_2_FBr [M+H]^+^ 463.0018 found: 463.0016

***N*-(4-Bromo-2-fluorophenyl)-6-methoxy-7-((1-methylpiperidin-4-yl)methoxy)quinazolin-4-amine^2, 12^ (Vandetanib, Caprelsa™)**

*N*-(4-Bromo-2-fluorophenyl)-6-methoxy-7-(piperidin-4-ylmethoxy)quinazolin-4-amine (30 mg, 0.065 mmol), sodium triacetoxyborohydride (19 mg, 0.091 mmol) was added to a flask containing formic acid (6 µL, 0.0845 mmol), acetic acid (5 µL, 0.078 mmol), DCM (1.6 mL) and methanol (3.2 mL). The flask was stirred for two hours at ambient temperature and then solvent removed under reduced pressure. Saturated NaHCO_3_ (3 mL) was added to the residue and the suspension filtered, washed with water (2 mL) and brine (2 mL). The filtrate was extracted with DCM (3 mL) and dried over MgSO_4_, solvent removed under reduced pressure, triturated with diethyl ether to afford the title compound as a white solid (6 mg, 84%).

Mp: 226-228 °C (lit 227-229 °C^12^); ^1^H NMR (500 MHz, *d_6_*_-_DMSO) δ: 1.44 (ddd, 2H, C*H*_2_, *J =* 3.9, 12.6, 24.8 Hz), 1.85 (d, 2H, C*H*_2_, *J =*12.5 Hz), 1.94-1.99 (m, 3H, C*H* and C*H*_2_), 2.32 (s, 3H, NC*H*_3_), 2.85 (d, 2H, C*H*_2_, *J =* 11.6 Hz), 3.98 (s, 3H, OC*H*_3_), 4.03 (d, 2H, OC*H*_2_, *J =* 5.5 Hz), 6.99 (s, 1H, Ar*H*), 7.21 (s, 1H, Ar*H*), 7.26 (s, 1H, Ar*H*), 7.34 (d, 1H, Ar*H*, *J =* 1.2 Hz), 7.36 (d, 1H, Ar*H*, *J =* 1.2 Hz), 8.51 (s, 1H, Ar*H*), 8.68 (brs, 1H, N*H*). ^13^C NMR (125 MHz, *d_6_*-DMSO) δ: 28.68, 34.92, 46.98, 49.32, 55.69, 70.65, 79.86, 102.82, 107.52 (d, *J =* 27.8), 108.91, 114.21, 114.74, 117.65, 121.21, 127.68, 131.32(d, *J =* 28.1 Hz), 153.04, 155.23, 155.99 (d, *J =* 132.3 Hz), 157.08, 158.86, 164.06. ^19^F NMR (471 MHz, *d_6_*-DMSO) δ: -115.86 (m, 1F). HRMS (ESI, m/z): calc’d for C_21_H_25_N_4_O_2_FBr [M+H]^+^ 462.1013 found: 462.1003

1. NMR

**4-Benzyloxy-3-methoxybenzonitrile (5)****
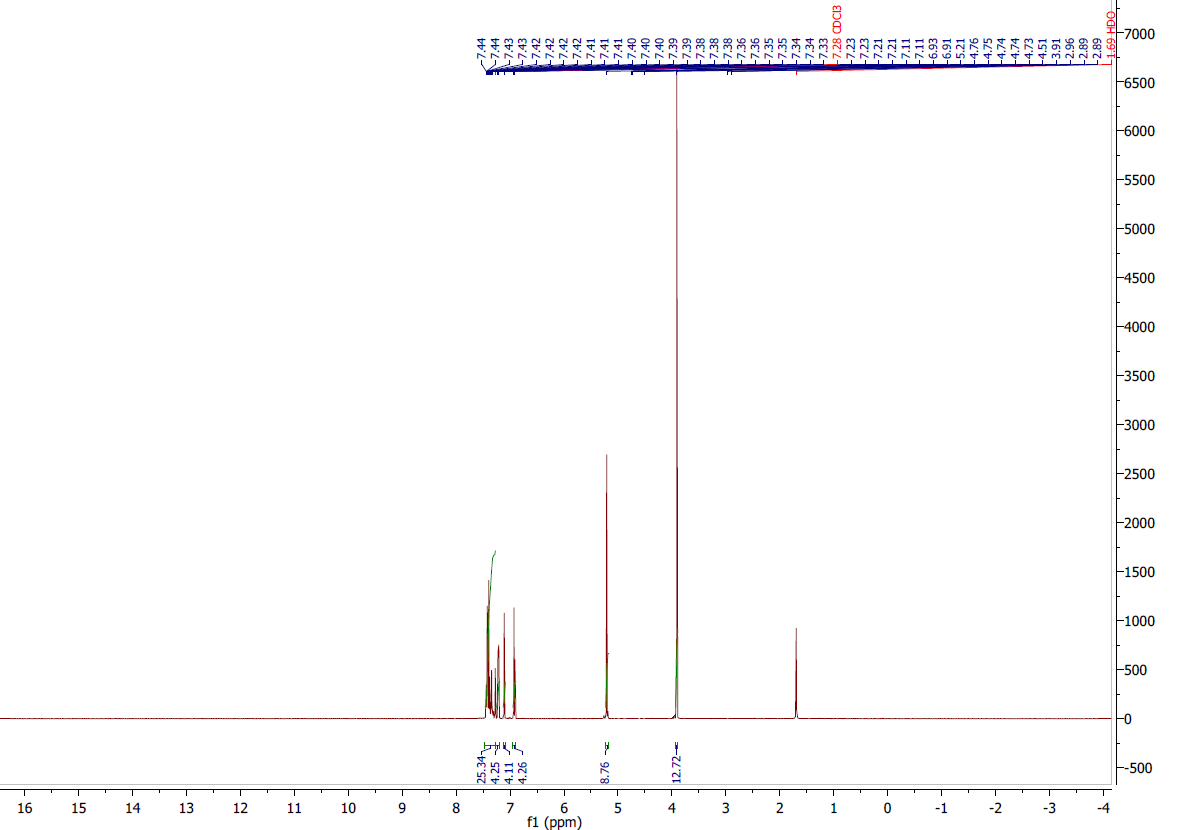
*****
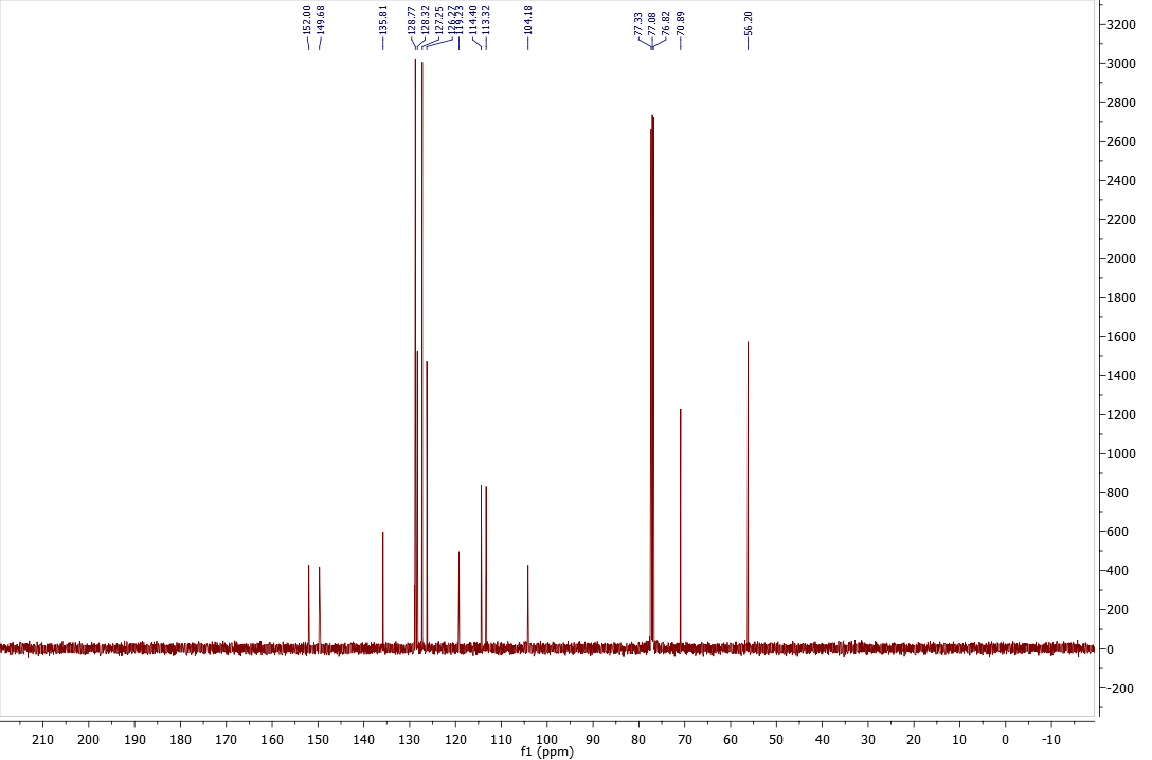
***

**4-Benzyloxy-5-methoxy-2-nitrobenzonitrile (6)**


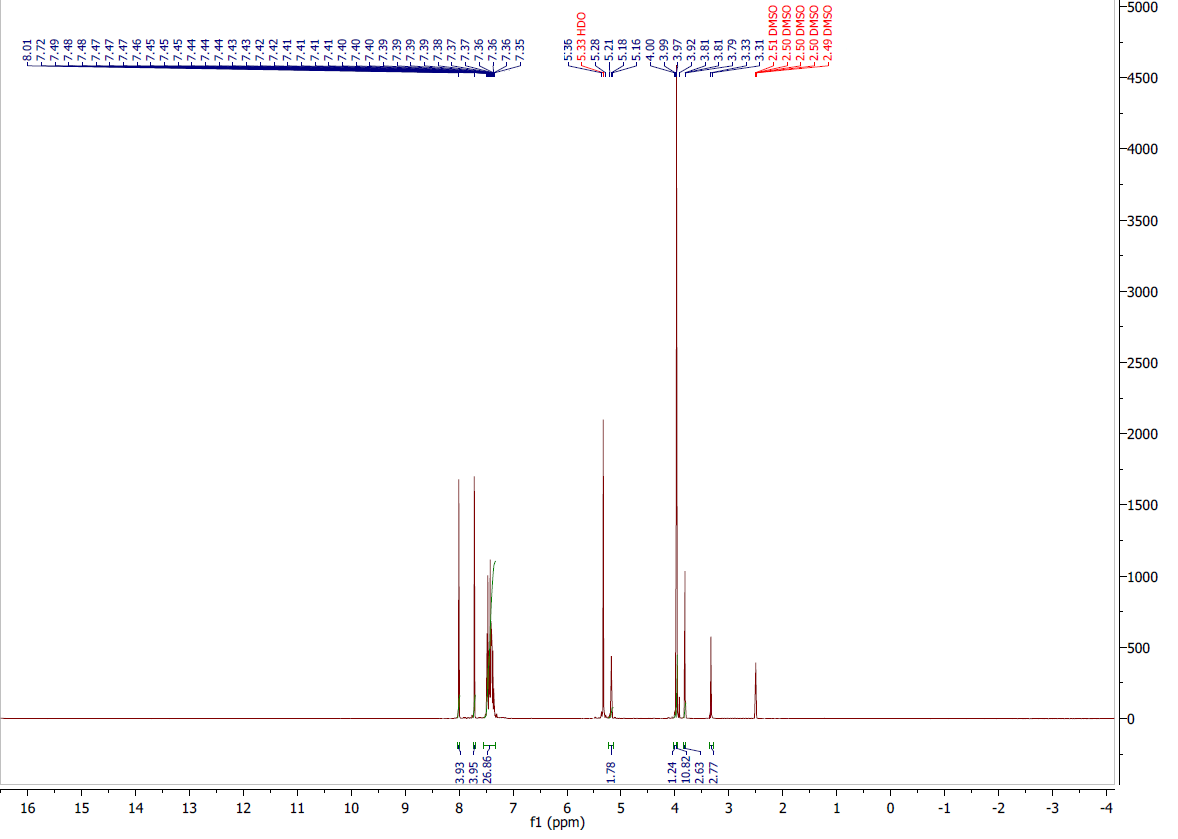

***
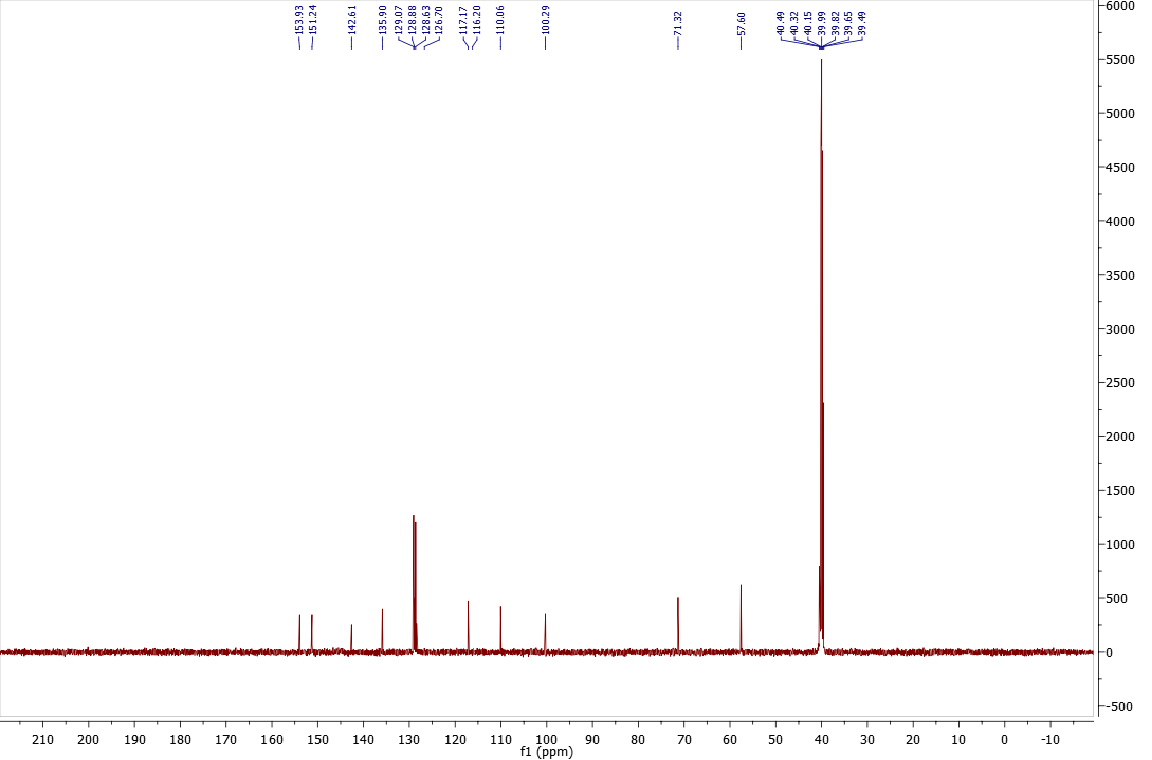
***

**2-Amino-4-benzyloxy-5-methoxybenzonitrile (7)**


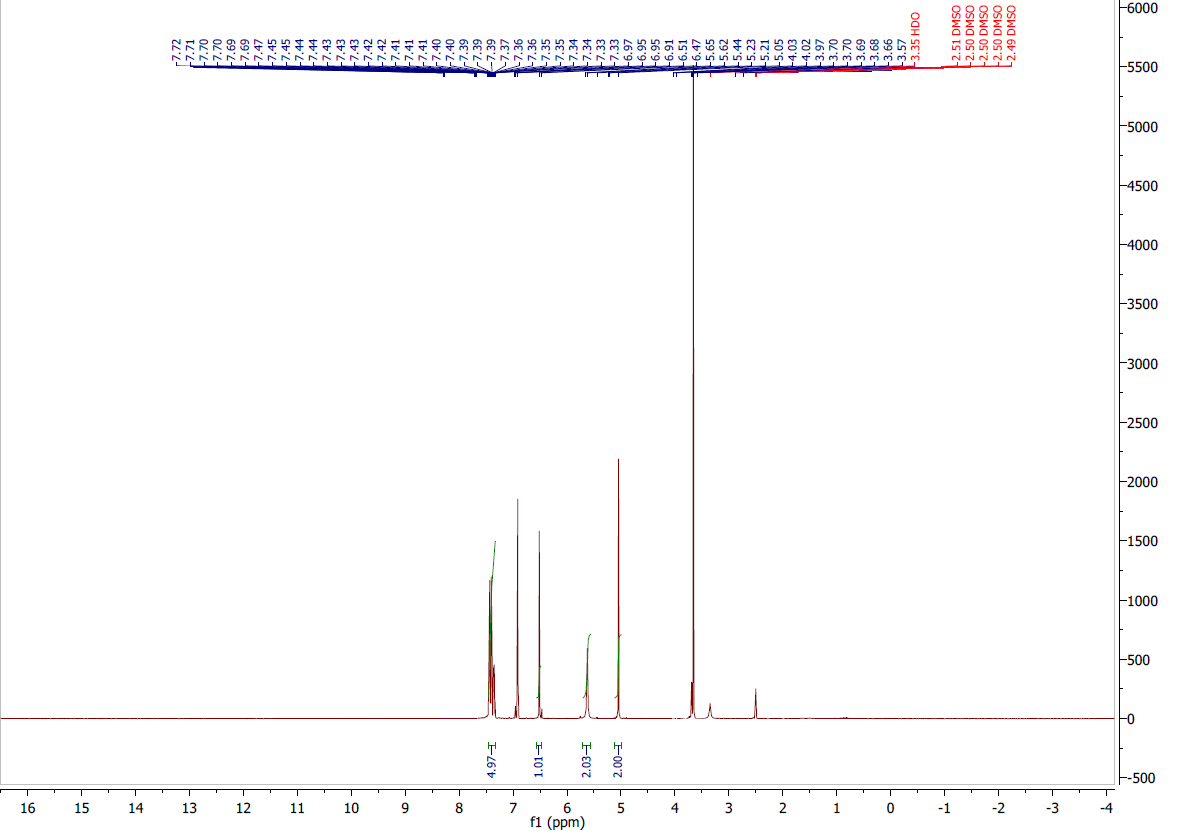

***
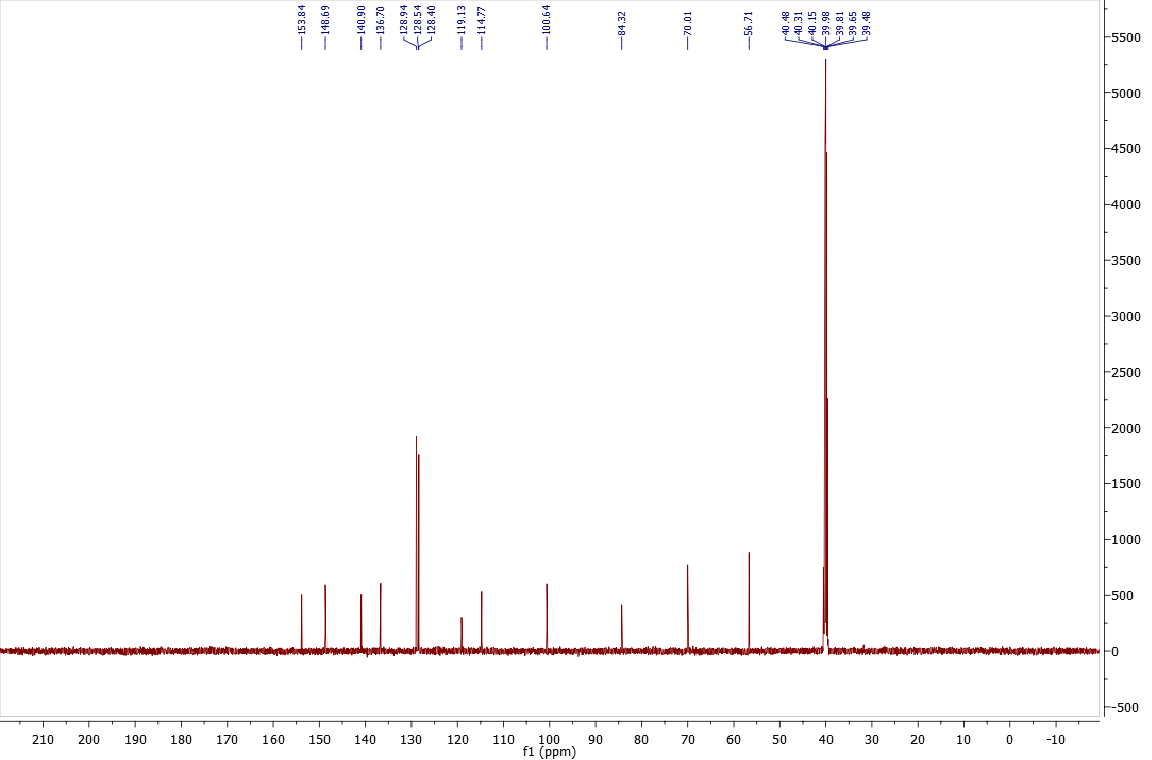
***

**(*E*)-*N*`-(5-Benzyloxy-2-cyano-4-methoxyphenyl)-*N,N*-dimethylformimidamide (8)**

**
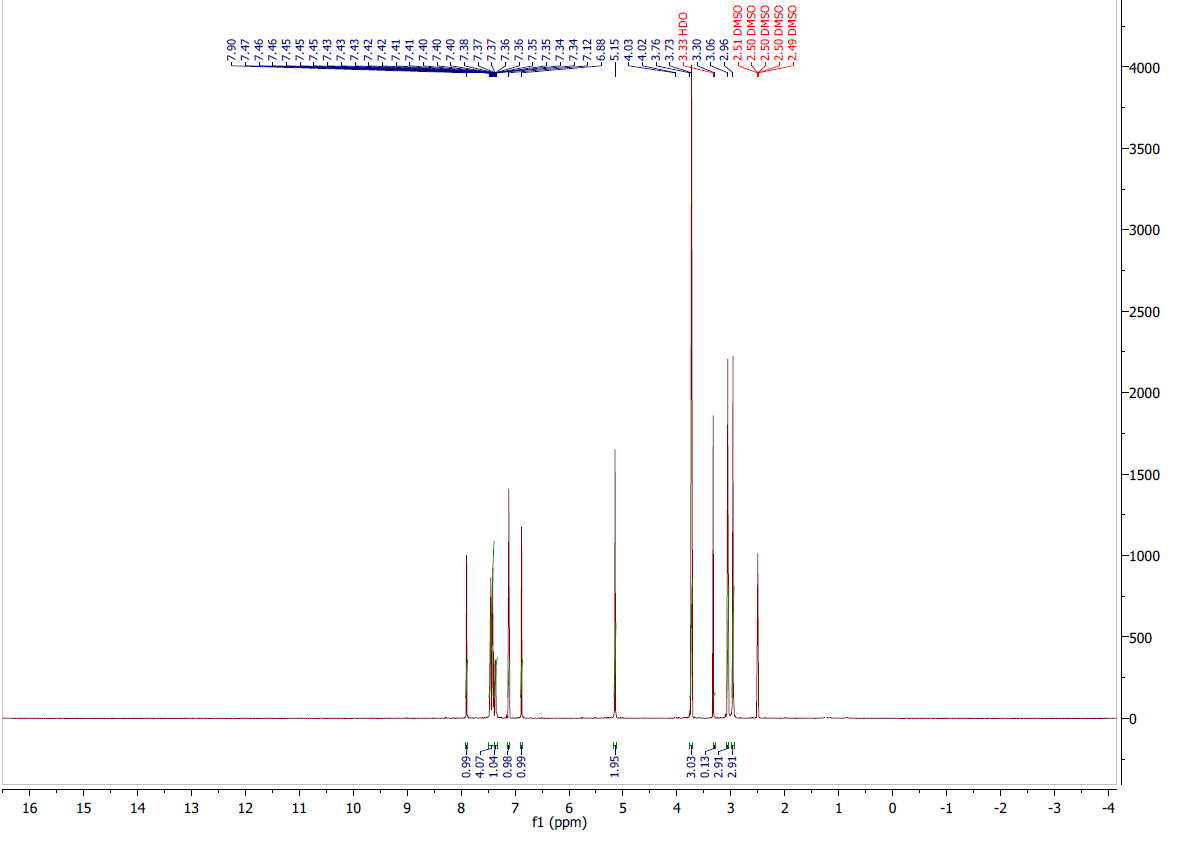
**

***
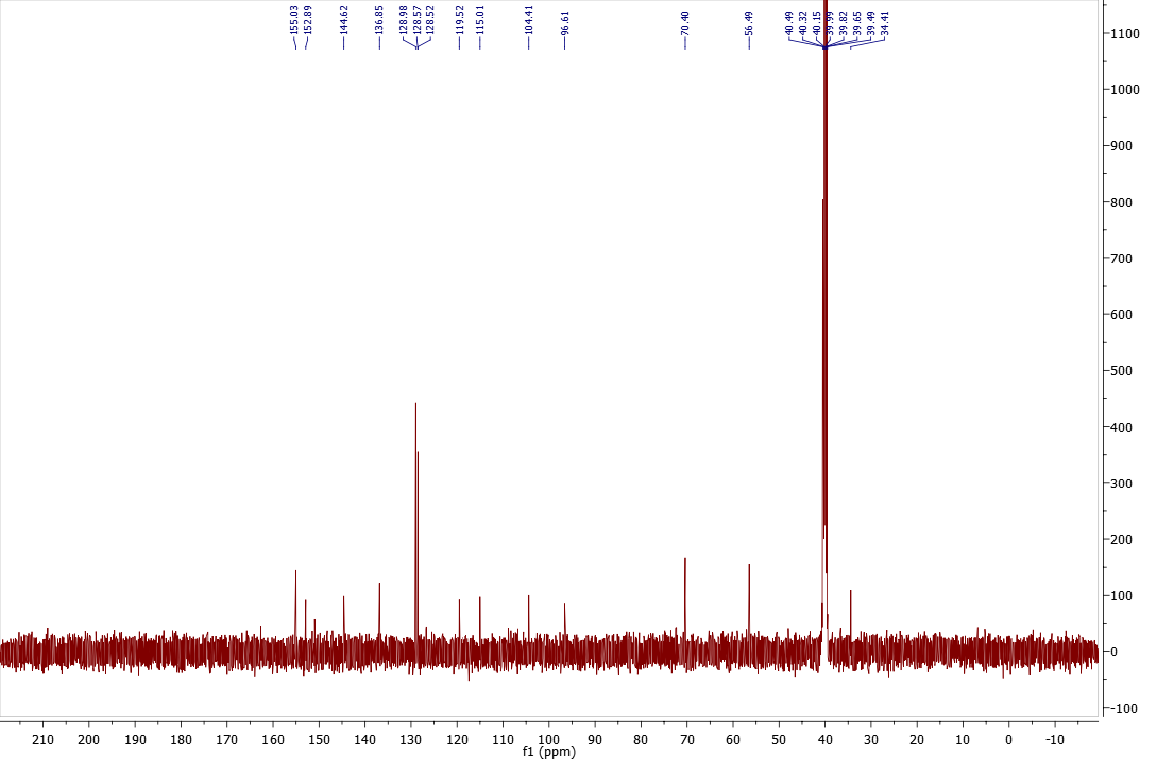
***

**(*E*)-*N*`-(2-Cyano-5-hydroxy-4-methoxyphenyl)-*N,N*-dimethylformimidamide (9)**

**
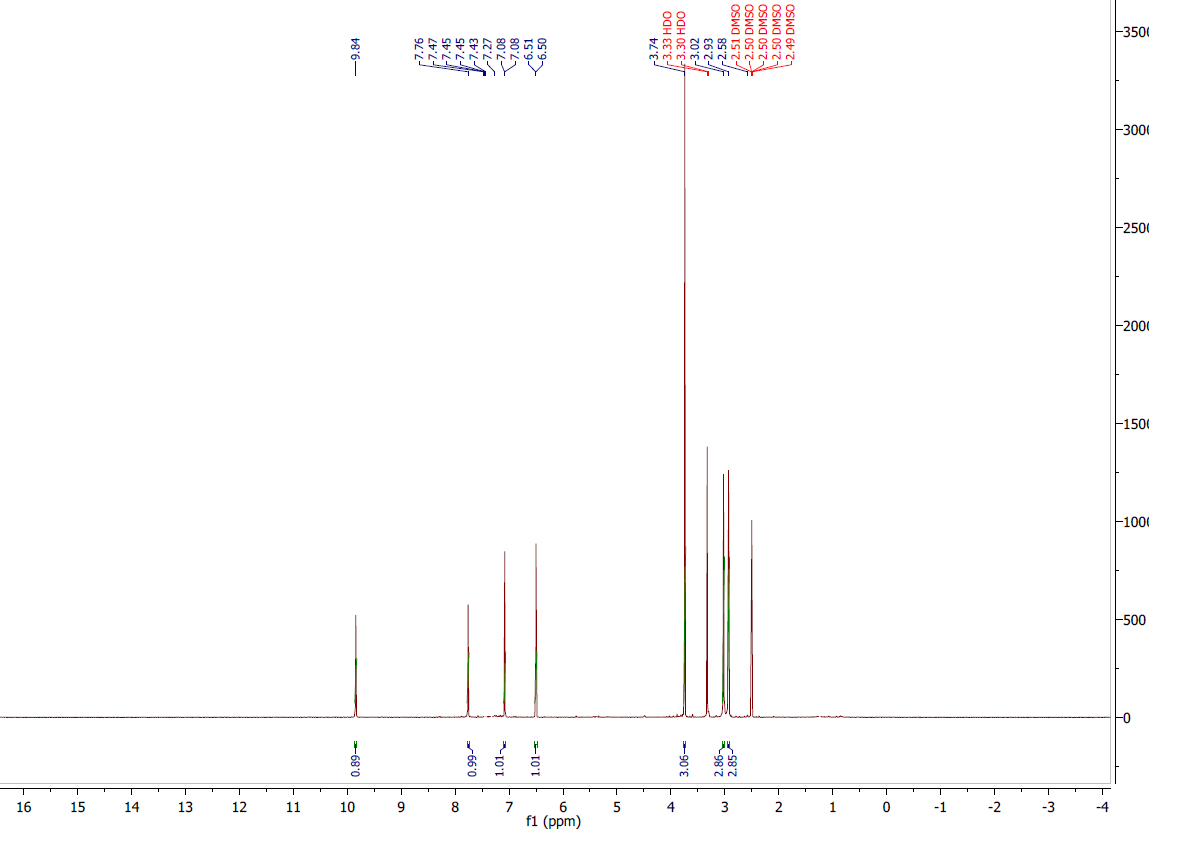
**

***
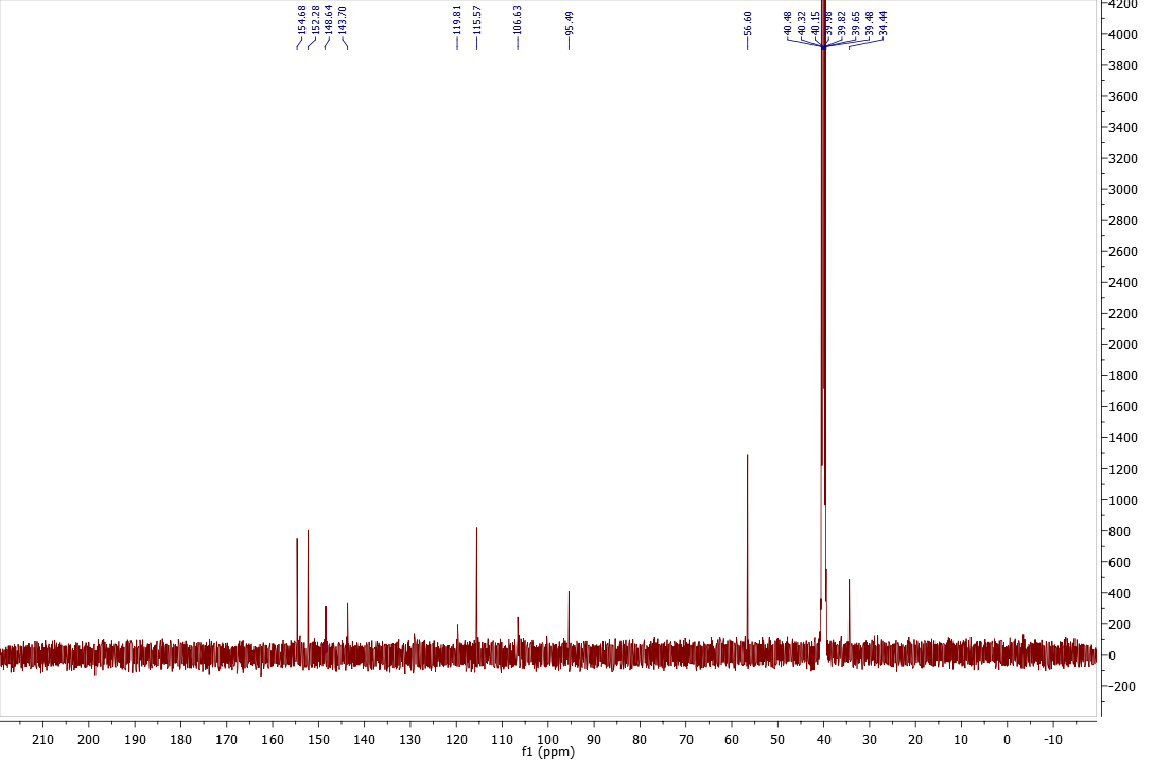
***

***tert*-Butyl-4-(tosyloxy)methyl)piperidine-1-carboxylate A**

***
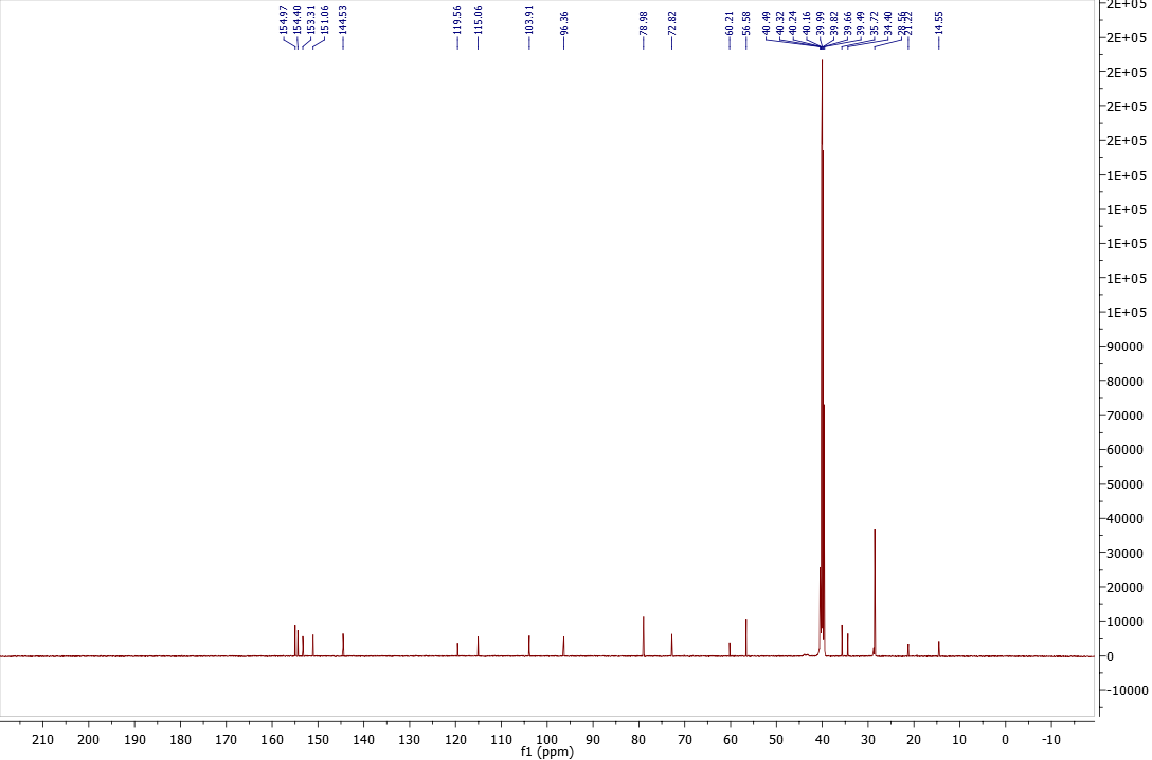
***

**(*E*)-*tert*-Butyl-4-((4-cyano-5-(((dimethylamino)methylene)amino)-2-methoxyphenoxy)methyl)piperidine-1-carboxylate (10)**

***
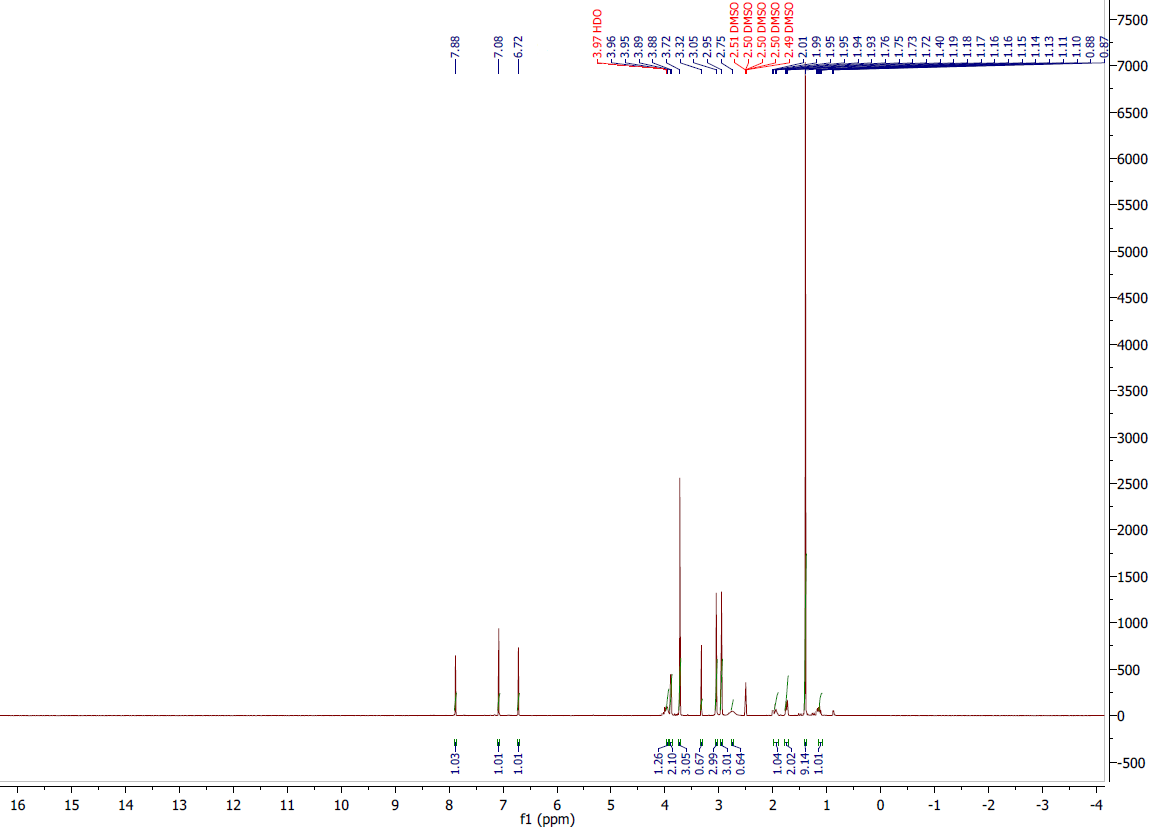
***

***
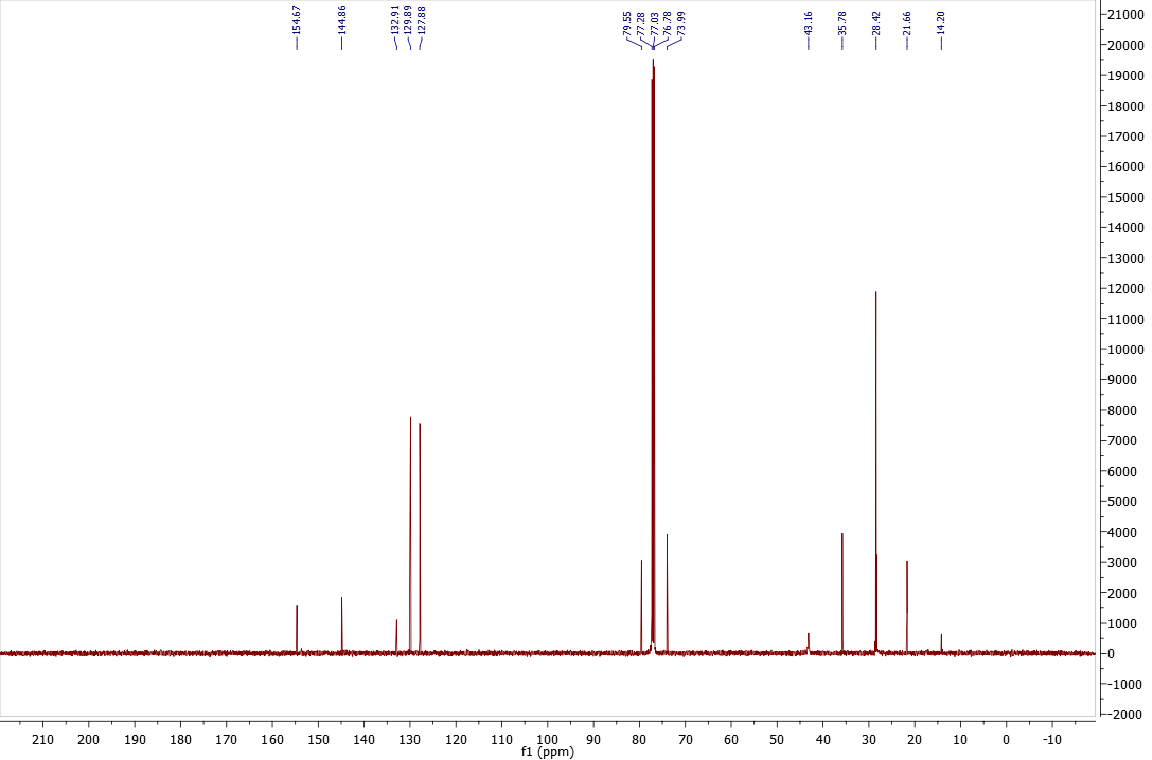
***

***tert*-Butyl 4-(((4-((4-bromo-2-fluorophenyl)amino)-6-methoxyquinazolin-7-yl)oxy)methyl)piperidine-1-carboxylate (11)**

***
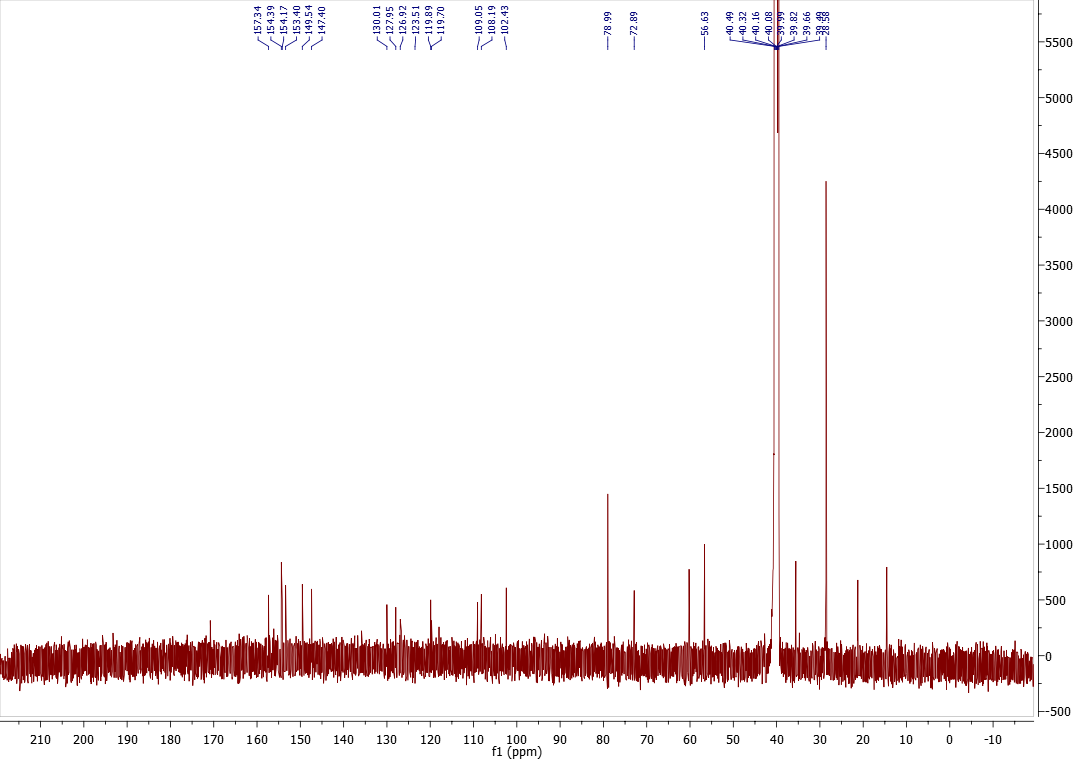
***

***N*-(4-Bromo-2-fluorophenyl)-6-methoxy-7-(piperidin-4-ylmethoxy)quinazolin-4-amine (12)****

***
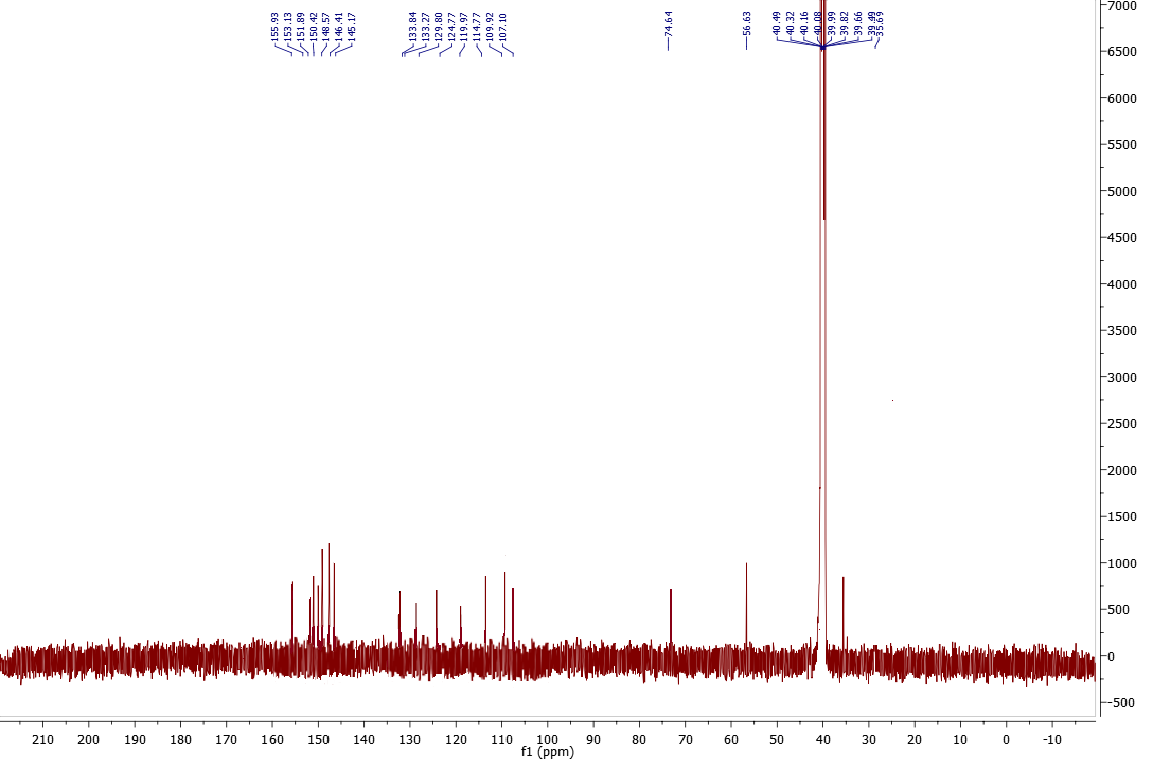
***

**References**

1 Althuis, T. H.; Hess, H. J. *J. Med. Chem.* **1977**, *20*, 146.

2 Hennequin, L. F.; Stokes, E. S. E.; Thomas, A. P.; Johnstone, C.; Plé, P. A.; Ogilvie, D. J.; Dukes, M.; Wedge, S. R.; Kendrew, J.; Curwen, J. O. *J. Med. Chem.* **2002**, *45*, 1300.

3 Hennequin, L. F.; Thomas, A. P.; Johnstone, C.; Stokes, E. S. E.; Plé, P. A.; Lohmann, J.-J. M.; Ogilvie, D. J.; Dukes, M.; Wedge, S. R.; Curwen, J. O.; Kendrew, J.; Lambert-van der Brempt, C. *J. Med. Chem.* **1999**, *42*, 5369.

4 Blixt, J.; Golden, M., David;; Hogan, P., John; ; Martin, D., Michael, Glanville; ; Montgomery, F., Joseph;; Patel, Z.; Pittam, J., David; ; Sependa, G., Joseph; ; Squire, C., John; ; Wright, N.; Cartwright, A. In *Chemical Process*, 2007; Vol. WO2007036713.

5 Liu, F.; Barsyte-Lovejoy, D.; Allali-Hassani, A.; He, Y.; Herold, J. M.; Chen, X.; Yates, C. M.; Frye, S. V.; Brown, P. J.; Huang, J.; Vedadi, M.; Arrowsmith, C. H.; Jin, J. *J. Med. Chem.* **2011**, *54*, 6139.

6 Fryatt, T.; Botting, N. P. *J. Labelled Compd. Radiopharm* **2005**, *48*, 951.

7 Heron, N. M.; Jung, J. H.; Pasquet, G. R.; Mortlock, A. A. In *Phosphonoxy quinazoline derivatives and their pharmaceutical use*; Astrazeneca, 2004; Vol. WO2004058781.

8 Liu, F.; Chen, X.; Allali-Hassani, A.; Quinn, A. M.; Wasney, G. A.; Dong, A.; Barsyte, D.; Kozieradzki, I.; Senisterra, G.; Chau, I.; Siarheyeva, A.; Kireev, D. B.; Jadhav, A.; Herold, J. M.; Frye, S. V.; Arrowsmith, C. H.; Brown, P. J.; Simeonov, A.; Vedadi, M.; Jin, J. *J. Med. Chem.* **2009**, *52*, 7950.

9 Jung, F. H.; Pasquet, G.; Lambert-van der Brempt, C.; Lohmann, J.-J. M.; Warin, N.; Renaud, F.; Germain, H.; De Savi, C.; Roberts, N.; Johnson, T.; Dousson, C.; Hill, G. B.; Mortlock, A. A.; Heron, N.; Wilkinson, R. W.; Wedge, S. R.; Heaton, S. P.; Odedra, R.; Keen, N. J.; Green, S.; Brown, E.; Thompson, K.; Brightwell, S. *J. Med. Chem.* **2006**, *49*, 955.

10 Mortlock, A. A. In *Quinazoline Compounds*, 2004; Vol. WO2004058752.

11 Galan, A.; Chen, J.; Du, H.; Forsyth, T.; Huynh, T. P.; Johnson, H. W. B.; Kearney, P.; Leahy, J. W.; Lee, M. S.; Mann, G.; Ridgway, B. H.; Takeuchi, C. S.; Zhou, P. In *Jak-2 modulators and methods of use*; Exelixis, 2008; Vol. WO2008042282

12 Gao, M.; Lola, C. M.; Wang, M.; Miller, K. D.; Sledge, G. W.; Zheng, Q. *Bioorg. Med. Chem. Lett.* **2011**, *21*, 3222.
